# Machine Learning Prediction of Intrapartum Cesarean Delivery in Women with Obesity

**DOI:** 10.3390/jcm15031125

**Published:** 2026-01-31

**Authors:** Daniel Gabbai, Itamar Gilboa, Roza Berkovitz Shperling, Lee Reicher, Emmanuel Attali, Yariv Yogev, Anat Lavie

**Affiliations:** 1 Department of Obstetrics and Gynecology, Lis Hospital for Women’s Health, Tel Aviv Sourasky Medical Center, Weizman 6, Tel Aviv 6423906, Israel; gabbaidaniel@gmail.com (D.G.); itamar.glb@gmail.com (I.G.); raz.bershp@gmail.com (R.B.S.); lee.reicher@gmail.com (L.R.); attaliemmanuel@gmail.com (E.A.); anatlavier@gmail.com (A.L.); 2 Gray Faculty of Medicine, Tel Aviv University, Tel Aviv 69978, Israel

**Keywords:** cesarean delivery, obesity, XGBoost, machine learning, intrapartum, prediction model, SHAP

## Abstract

**Objective**: To identify risk factors for intrapartum cesarean delivery (CD) among women with obesity (BMI ≥ 30) and to evaluate whether a machine learning model (XGBoost) can improve prediction of this outcome compared with a previously developed regression-based risk score. **Methods**: A retrospective cohort study at a single university-affiliated tertiary medical center was conducted. All women with a pre-pregnancy BMI ≥ 30 who initiated a trial of labor between 2012 and 2024 were included. Women who underwent elective CD or had missing outcome data were excluded. Maternal, obstetric, and intrapartum characteristics were compared between women who delivered vaginally and those who required an intrapartum CD. Predictors were evaluated using extreme gradient boosting (XGBoost), and model performance was assessed using receiver operating characteristic (ROC) analysis and SHAP-based interpretability. **Results**: Among 146,999 women who delivered during the study period, 10,248 (7.0%) had a pre-pregnancy BMI ≥ 30. A total of 7236 obese women attempted a trial of labor, of whom 1031 (14.5%) underwent an intrapartum CD. Key predictors included limited cervical dilatation at admission, epidural anesthesia, nulliparity, maternal BMI and age, oxytocin use, birthweight, inflammatory markers (white blood count and neutrophils to lymphocytes ratio), and previous cesarean scar. The XGBoost model demonstrated excellent discriminatory ability with an AUC of 0.945 (95%CI 0.930–0.960, DeLong), and exceeded the performance of our previous regression-based score, and provided detailed insight into nonlinear effects through SHAP analysis. In a secondary analysis restricted to variables available at admission, a pre-labor model retained a strong discriminatory performance across BMI categories, supporting its applicability for early risk stratification prior to labor onset. **Conclusions**: A machine learning-based model accurately predicts intrapartum cesarean delivery in women with obesity and may serve as a valuable tool to support individualized counseling and delivery planning.

## 1. Introduction

Obesity has become one of the most prominent health challenges affecting women of reproductive age, with rates increasing steadily worldwide [1,2]. As body mass index (BMI) rises, so does the risk for antepartum, intrapartum, and post-partum complications [3,4,5]. One of the most clinically significant consequences is increased likelihood of cesarean delivery (CD), particularly unplanned intrapartum CD [6].

Intrapartum CD is associated with substantial short- and long-term maternal risks [7], which tend to be further amplified in women with obesity [8,9,10]. The combination of higher baseline morbidity and an increased probability of requiring an unscheduled surgical intervention underscores the need for accurate, individualized risk estimation prior to labor. Although previous research has identified several clinical factors associated with intrapartum CD among women with elevated BMI, available prediction tools remain limited in scope and performance [11,12,13].

In a prior study [14], we constructed a clinical risk score for intrapartum CD using conventional regression methods in a large cohort of women with obesity attempting vaginal birth. While that approach yielded a practical bedside assessment tool, modern analytical techniques may offer improved precision. Machine learning algorithms can detect nonlinear effects and higher order interactions that traditional statistical models may overlook.

In the current study, we aimed to apply Extreme Gradient Boosting (XGBoost) to predict intrapartum CD among women with BMI ≥ 30, and to compare its performance with the previously developed conventional regression-based model.

## 2. Methods

### 2.1. Study Population

We conducted a retrospective cohort study of all women who planned a trial of labor at a single, university-affiliated tertiary medical center, between 2012–2024. We identified all women with a pre-pregnancy BMI ≥ 30 kg/m^2^. Consistent with our previous study [14], only women who initiated labor with the intention of a vaginal delivery were eligible for inclusion. Women who underwent planned elective CD, had missing data for BMI or outcome data, had contraindication to a trial of labor or lacked information on the mode of delivery were excluded.

Among eligible women with BMI ≥ 30, we classified the study outcome as intrapartum CD. The comparison group included women who achieved either a spontaneous or an assisted vaginal birth. The final cohort represented a similar population evaluated in our previous risk score model, allowing for a direct comparison of predictive performance between the two analytical approaches.

### 2.2. Statistical Analysis

Baseline maternal and intrapartum characteristics were compared between women who completed a vaginal delivery and those who underwent intrapartum CD. Continuous variables were assessed for normality within each delivery group using the Shapiro–Wilk test. Normally distributed variables were summarized as mean ± standard deviation and compared using the independent *t*-test, whereas non-normally distributed variables were presented as median with interquartile range and compared using the Wilcoxon rank-sum test. Categorical variables were reported as counts with percentages and compared using the chi-square test or Fisher’s exact test when appropriate.

As described in our prior publication, a multivariable logistic regression model was previously constructed to identify independent risk factors for intrapartum CD among women with obesity and to derive a clinical risk score. The prior model served as the baseline reference for performance comparison in the present analysis.

For the current study, we applied XGBoost to our cohort to evaluate whether a machine learning approach improves prediction of intrapartum CD. To specifically address the clinical need for risk estimation prior to labor, model development was restricted to variables available at admission, before the onset of active labor. Accordingly, intrapartum and post-delivery variables were excluded from model training. This pre-labor model was designed to reflect real-world decision-making at the time of admission, when clinicians must determine the likelihood of successful vaginal delivery before labor progression occurs.

The dataset was randomly divided into training (70%) and testing (30%) sets using stratification by outcome. No temporal separation was applied. To prevent information leakage, missing values were imputed using parameters derived exclusively from the training set and subsequently applied to the test set. Categorical variables were encoded using factor levels defined in the training set and consistently applied to the testing set. Given the imbalance between vaginal and cesarean deliveries, class weighting was applied using a scale_pos_weight parameter derived from the training data. The XGBoost model was trained using a binary logistic objective function with predefined hyperparameters, including maximum tree depth, learning rate, subsampling fraction, and column subsampling fraction. Model performance was evaluated on the independent test set using receiver operating characteristic (ROC) analysis, with calculation of the area under the curve (AUC) and 95% confidence intervals. 

To provide transparent and clinically interpretable insights into the contribution of each predictor, we applied SHapley Additive exPlanations (SHAP) values to the final XGBoost model. SHAP values quantify both the magnitude and direction of each variable’s contribution to the predicted probability of intrapartum CD, allowing visualization of how increasing or decreasing values of a given feature influence model output. In this framework, positive SHAP values indicate an increased likelihood of CD, whereas negative values indicate a protective effect. This approach enables clinicians to interpret complex, non-linear relationships in an intuitive manner and to understand how individual patient characteristics contribute to overall risk estimates.

All analyses were performed using R version 4.5.1, with the packages xgboost, pROC, dplyr, tidyr, shapviz, and ggplot2.

### 2.3. Data Collection

All data were extracted from the institutional electronic delivery-room database, which prospectively records demographic, obstetric, laboratory, and intrapartum information for all parturient. The dataset included maternal characteristics such as age, pre-pregnancy BMI, gestational weight gain, parity, gravidity, and obstetric history. Clinical and pregnancy-related conditions were also collected, including gestational diabetes, pre-gestational diabetes, pre-eclampsia, smoking status, twin gestation, and intrauterine fetal demise.

Intrapartum variables included the mode of labor initiation, premature rupture of membranes, cervical dilatation at admission, use of regional anesthesia, administration of oxytocin, development of intrapartum fever (≥38 °C) and additional labor characteristics. Laboratory results were obtained from the last complete blood count performed prior to delivery including hemoglobin concentration, platelet count, white blood cell count (WBC), and the neutrophil-to-lymphocyte ratio (NLR). Neonatal information included birthweight and fetal sex.

All variables were collected and processed uniformly across the study period. Missing values were handled through median imputation for continuous variables and mode imputation for categorical variables, in accordance with standard preprocessing requirements for gradient-boosting models.

The study was approved by the institutional review board (IRB TLV-0284-08, 2025). Data analysis was performed with an unidentified database. Therefore, informed consent was not needed.

## 3. Results

During the study period, a total of 146,999 women delivered at our center. Of these, 10,248 women (7.0%) had a pre-pregnancy BMI ≥ 30. After excluding women with planned CD, missing key data, or contraindications to a trial of labor, 7236 obese women remained eligible for analysis. Among them, 6187 women (85.5%) achieved a vaginal birth, while 1031 women (14.5%) required an intrapartum CD.

Table 1 presents a comparison of demographic, laboratory, obstetric, and intrapartum characteristics between women who delivered vaginally and those who required intrapartum CD. Women who underwent CD were slightly older (median 34.3 vs. 32.7 years, *p* < 0.001) and had a marginally higher pre-pregnancy BMI (33.2 vs. 32.6 kg/m^2^, *p* < 0.001). Cervical dilatation at admission was notably lower among women who ultimately required CD (*p* < 0.001). Nulliparity showed a substantial difference between groups (59.6% vs. 37.7%, *p* < 0.001), and previous CD was markedly more common in the CD group (19.0% vs. 4.8%, *p* < 0.001). Medical comorbidities also differed: gestational diabetes was more prevalent among women who underwent CD (33.4% vs. 25.3%, *p* < 0.001), as were preeclampsia (10.5% vs. 2.9%, *p* < 0.001) and twin gestations (15.8% vs. 1.6%, *p* < 0.001). Several intrapartum features were strongly associated with CD, including oxytocin use (58.7% vs. 53.9%, *p* = 0.0045) and intrapartum fever (12.7% vs. 2.6%, *p* < 0.001). Continuous laboratory parameters such as NLR, WBC and gestational weight gain also demonstrated statistically significant differences (*p* < 0.05 for all). Birthweight was substantially lower in the CD group (3150 vs. 3355 g, *p* < 0.001).

**Figure 1:**
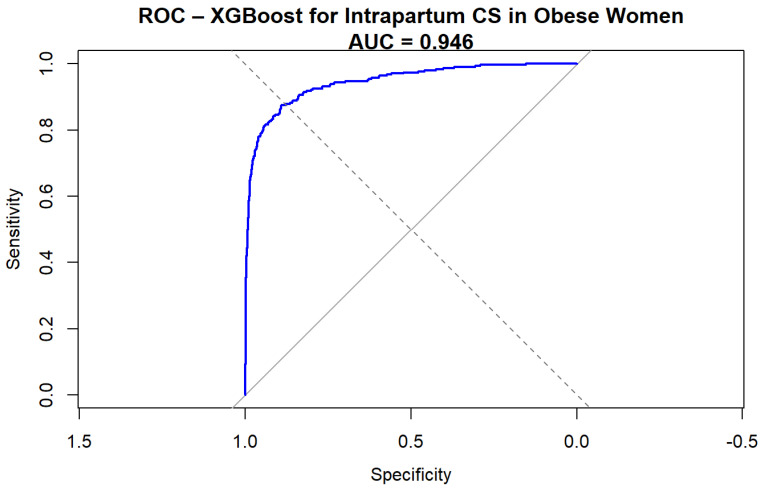
ROC curve for XGBoost prediction of intrapartum cesarean delivery.

The Extreme Gradient Boosting (XGBoost) model demonstrated excellent discrimination for predicting intrapartum cesarean delivery among obese women. The ROC curve (Figure 1) yielded an AUC of 0.94 (95%CI 0.93–0.96, DeLong), indicating a very high predictive performance.

**Figure 2:**
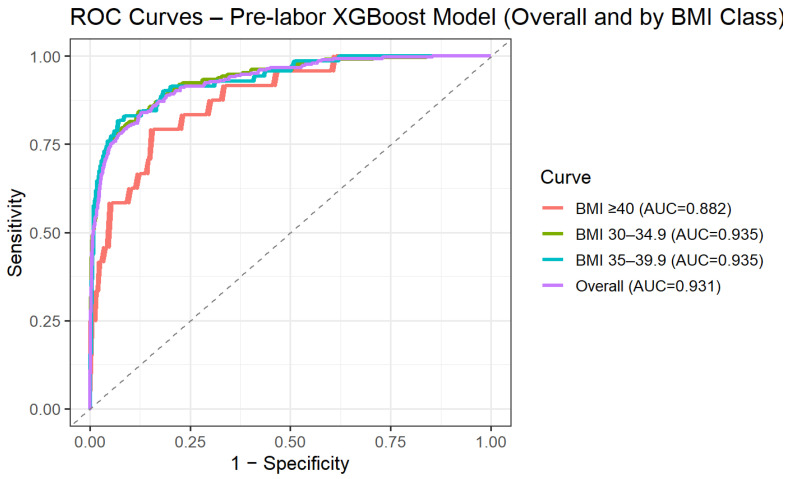
ROC curve for XGBoost pre-labor prediction of intrapartum cesarean delivery by BMI categories.

To address the clinical need for risk assessment prior to the onset of labor, we performed a secondary analysis restricted to variables available at admission, excluding intrapartum and post-delivery parameters. Using this pre-labor model, predictive performance remained high, with an AUC of 0.93 (95% CI 0.92–0.95) on the independent test set. Model performance was further evaluated across BMI categories. Discrimination remained stable across obesity subclasses, with AUCs of 0.94 (95% CI 0.92–0.95) for BMI 30.0–34.9, 0.94 (95% CI 0.90–0.97) for BMI 35.0–39.9, and 0.88 (95% CI 0.81–0.95) for BMI ≥ 40 (Figure 2). These findings suggest that the predictive ability of the model is preserved across increasing degrees of obesity.

When evaluated on the held-out test set, the model showed strong classification performance across clinically relevant thresholds. At a probability threshold of 0.20, chosen to maximize sensitivity for screening purposes, sensitivity was 93.5% and specificity was 73.7%, with a positive predictive value (PPV) of 37.0% and a negative predictive value (NPV) of 98.6%. Using the Youden-optimized threshold (0.462), sensitivity was 84.6% and specificity was 91.0%, with a PPV of 60.8% and an NPV of 97.3%.

**Figure 3:**
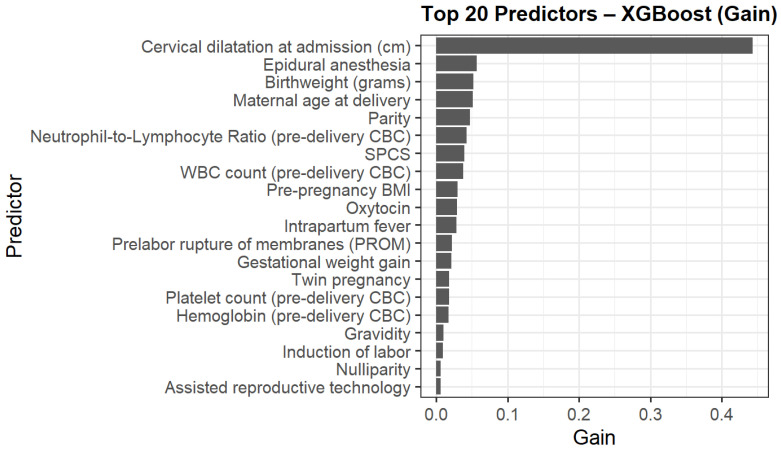
Top 20 predictors of intrapartum cesarean delivery ranked by XGBoost model gain.

The top contributors to model gain are presented in Figure 3. Cervical dilatation at admission was the strongest predictor, followed by epidural anesthesia, birthweight, maternal age delivery, parity, NLR and WBC. Additional contributors included pre-pregnancy BMI, previous one low transverse cesarean section (SPCS), oxytocin use, and intrapartum fever.

**Figure 4:**
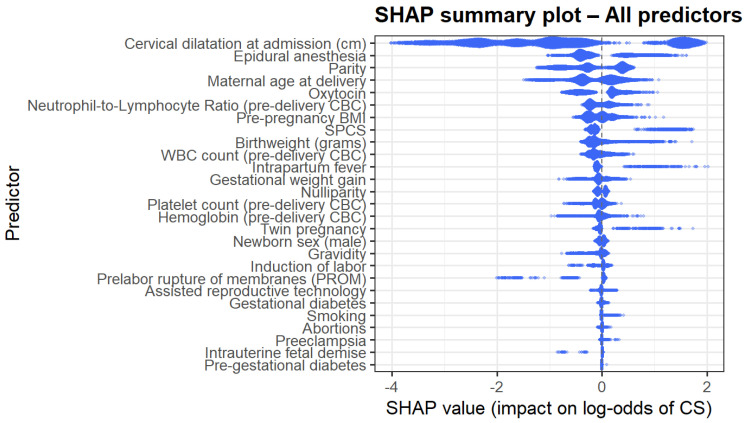
SHAP summary plot.

The SHAP summary plot (Figure 4) provides a global interpretation of model behavior. Predictors with the largest absolute impact on the log odds of CD mirrored the gain-based ranking; cervical dilatation, epidural anesthesia, maternal age, NLR, birthweight, parity, and inflammatory markers demonstrated the most substantial influence. The directionality of effects was nonlinear and often complex, supporting the use of machine learning methods to model such complex interactions.

**Figure 5:**
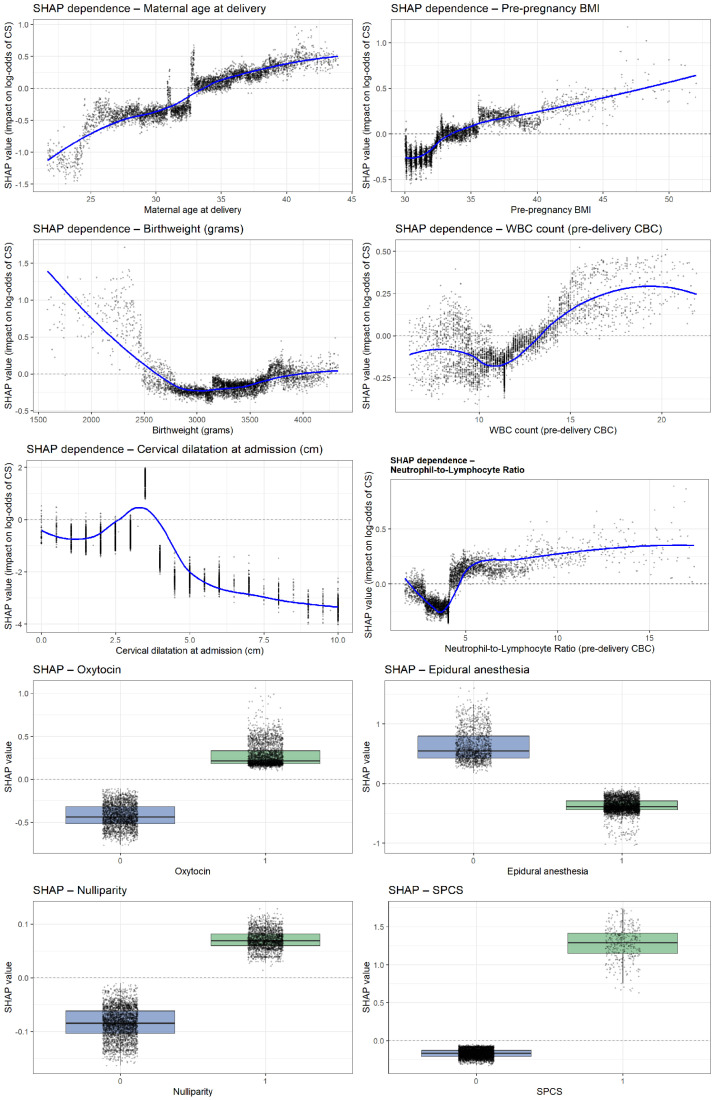
SHAP dependence plots for the top predictors of intrapartum cesarean delivery.

Analysis of the ten most influential predictors revealed several consistent nonlinear patterns shaping the model’s estimation of intrapartum cesarean risk (Figure 5). Very early cervical dilatation at admission (0–2 cm) showed the strongest effect, while progressive dilatation markedly reduced risk. Use of epidural anesthesia, oxytocin administration, and nulliparity each demonstrated clear upward shifts in SHAP values, indicating substantial contributions to cesarean likelihood. Higher pre-pregnancy BMI and increasing maternal age were both associated with progressively elevated risk. Inflammatory biomarkers exhibited parallel nonlinear trends: both the NLR and WBC showed higher predicted risk beyond thresholds of approximately 4.5 and 13–14 × 10^9^/L, respectively. Birthweights displayed a pronounced U-shaped relationship, with markedly higher risk at very low birthweights and a gradual rise again. Finally, SPCS emerged as one of the strongest categorical predictors, consistently shifting SHAP values toward a higher probability of intrapartum cesarean delivery.

## 4. Discussion

### 4.1. Principle Findings

In this study, we evaluated predictors of intrapartum CD among women with BMI ≥ 30 and assessed whether a machine learning model could enhance prediction. The main findings of our study include: (a) Intrapartum CD occurred at a relatively high rate among women with BMI ≥ 30 attempting vaginal birth. (b) Several factors were strongly associated with intrapartum CD, including low cervical dilatation at admission, epidural anesthesia, nulliparity, higher maternal BMI and age, oxytocin use, birthweight, elevated inflammatory markers, and previous cesarean scar. (c) The XGBoost model achieved an excellent predictive performance and, when evaluated on a similar set, outperformed our previously developed regression-based clinical risk score. Importantly, the model was designed as a dynamic intrapartum prediction tool rather than a static admission-based score, incorporating variables that evolve during labor. Accordingly, variables such as epidural analgesia or oxytocin administration should be interpreted as markers of evolving labor dynamics rather than direct causal determinants of CD, reflecting real-time clinical decision-making rather than baseline risk alone.

### 4.2. Our Findings in the Context of Other Observations

Several of the strongest predictors identified in our XGBoost model align closely with existing evidence regarding intrapartum CD among women with obesity. Increasing pre-pregnancy BMI has repeatedly been linked to higher intrapartum CD rates, with prior studies demonstrating a progressive rise in risk across obesity classes [15]. Our model reproduced this pattern, showing a steadily increasing SHAP contribution beyond a BMI of about 38 kg/m^2^.

The strong influence of nulliparity [16] and maternal age [17,18] is consistent with earlier reports describing both factors as major contributors to failed labor progression among women with obesity. Similarly, elevated inflammatory markers, including WBC and the NLR, have been associated with impaired uterine contractility and higher CD rates in observational studies [19,20]. We observed analogous trends, with higher NLR and WBC values associated with an increased predicted risk of intrapartum CD, consistent with threshold effects reported in prior literature. Notably, SHAP analysis revealed a non-linear relationship between inflammatory markers and cesarean risk, suggesting that heightened inflammatory burden may contribute to impaired labor progression. These findings are biologically plausible and hypothesis-generating, although causality cannot be inferred from the present analysis. Finally, cervical dilatation at admission emerged as the most influential predictor in our model, reflecting baseline labor status and supporting prior evidence that early admission with limited cervical dilatation is associated with an increased likelihood of intrapartum cesarean delivery [21,22].

Oxytocin augmentation and epidural anesthesia, both strong contributors in our SHAP analysis, have consistently been linked to prolonged labor and higher rates of surgical intervention in this population [23,24]. Prior studies describe reduced uterine contractility, increased oxytocin requirements, and greater likelihood of labor arrest among women with elevated BMI [25], all of which correspond with the risk patterns we observed. Notably, although epidural use appeared more common among women who ultimately delivered vaginally in our unadjusted Table 1, this finding likely reflects the underlying structure of the cohort: the vaginal delivery group was substantially larger, and many intrapartum cesarean deliveries occurred before an epidural could be administered. After adjusting clinical and obstetric factors in the multivariable model, epidural anesthesia behaved as expected, emerging as an independent predictor of intrapartum CD, fully aligned with previous reports.

Previous prediction tools for intrapartum CD in women with obesity have relied primarily on logistic regression or simpler machine learning models, generally achieving modest discrimination (AUCs of 0.70–0.85 in most reports) [13,14]. Notably, prior ML-based studies have often focused on restricted populations, such as nulliparous women or those with class III obesity, limiting their generalizability. In contrast, our XGBoost model was developed in a broader obstetric population encompassing all obesity classes (BMI ≥ 30) and integrated both clinical and hematologic variables, achieving a superior discriminatory performance (AUC 0.95, 95% CI 0.93–0.96). Beyond overall performance, our model offers additional value through the use of SHAP-based interpretability, which enables visualization of non-linear effects and individualized risk contributions. In particular, the identification of threshold effects for inflammatory markers such as WBC and NLR provides biologically plausible insight into the relationship between inflammatory burden and labor progression. While direct statistical comparison with previously published models is limited by differences in study populations and design, these findings suggest that machine learning-based approaches may offer incremental predictive and interpretive advantages over traditional regression-based tools, particularly for real-time intrapartum risk assessment.

### 4.3. Clinical Implications

CD has become increasingly common, paralleling the rising prevalence of obesity among women of reproductive age. Among women with BMI ≥ 30, intrapartum CD is associated with increased perioperative and postoperative morbidity, particularly when performed emergently. To address the clinical need for accurate pre-labor risk stratification, we therefore developed and evaluated a secondary prediction model restricted exclusively to variables available at the time of admission, prior to labor progression. This pre-labor model excluded intrapartum and post-delivery variables, including birthweight, and was designed to reflect real-world clinical decision-making when counseling patients regarding trial of labor.

Although the full dynamic model demonstrated a superior overall predictive performance, the pre-labor model retained strong discriminatory ability, supporting its clinical relevance for early risk assessment. The dynamic XGBoost model was subsequently constructed to capture evolving intrapartum information, incorporating both clinical and laboratory variables obtained at admission and during labor. In this context, the model enables identification of women at increased risk for intrapartum CD, such as those presenting with limited cervical dilatation in combination with nulliparity, elevated inflammatory markers, or a prior uterine scar. Importantly, the model is intended to support clinical awareness and risk stratification rather than dictate intervention, and its SHAP-based interpretability facilitates transparent communication of key contributors to risk. Together, these findings highlight the potential role of the model as a clinical decision-support tool to aid intrapartum monitoring and preparedness, pending external validation.

### 4.4. Strengths and Limitations

Our study possesses several strengths. It uses a large and contemporary cohort of obese women attempting vaginal birth, allowing a detailed evaluation of risk factors relevant to this population. Importantly, unlike prior studies that relied solely on traditional regression approaches, we applied a machine learning framework capable of capturing nonlinear effects and higher order interactions, resulting in an improved predictive performance and clearer insight into variable contributions through SHAP analysis. The use of SHAP analysis partially overcomes the ‘black box’ nature of machine learning models by providing clinically interpretable explanations at both the global and individual-patient level.

Nonetheless, several limitations must be acknowledged. First, as a retrospective single-center cohort study spanning more than a decade, the findings may be influenced by local practice patterns, patient case-mix, and temporal changes in obstetric management, which may limit generalizability to other settings. Although this design ensures internal consistency, external and temporal validation across diverse institutions and healthcare systems is required before broad clinical implementation. Reliance on computerized chart documentation also renders the analysis susceptible to information bias. Moreover, we did not evaluate model calibration (e.g., calibration-in-the-large/slope or calibration plots) or perform decision curve analysis, which are important for translating predictive performance into clinical utility. In addition, we did not assess long-term maternal outcomes such as pelvic floor dysfunction, urinary symptoms, or postpartum sexual health, limiting our ability to evaluate broader consequences of delivery mode decisions. External validation in independent populations, as well as validation across different time periods, is necessary before clinical implementation.

## 5. Conclusions

In our study of women with BMI ≥ 30 attempting vaginal delivery, intrapartum CD occurred at a substantial rate and was strongly associated with multiple clinical, obstetric, and inflammatory factors. The XGBoost model demonstrated excellent predictive accuracy and outperformed our previously developed regression-based score. Incorporating the machine learning-derived risk estimation into pre-labor counseling may enhance shared decision-making and support individualized delivery planning. Further external validation and assessment of performance over time are needed to confirm these findings and to refine the model for clinical use.

## Figures and Tables

**Table 1:** Maternal, obstetric, intrapartum, and neonatal characteristics stratified by mode of delivery among obese women attempting a trial of labour.

Variable	Vaginal Delivery (*n* = 6187)	CD (*n* = 1031)	*p*-Value
Maternal age at delivery, median (IQR)	32.7 (29.4–36.2)	34.3 (30.7–38.0)	<0.0001
Pre-pregnancy BMI, median (IQR)	32.6 (31.1–35.1)	33.2 (31.2–35.9)	<0.0001
Previous CD, *n* (%)	298 (4.8%)	196 (19.0%)	<0.0001
Nulliparity, *n* (%)	2334 (37.7%)	614 (59.6%)	<0.0001
Gestational diabetes, *n* (%)	1567 (25.3%)	344 (33.4%)	<0.0001
Preeclampsia, *n* (%)	182 (2.9%)	108 (10.5%)	<0.0001
Twin pregnancy, *n* (%)	96 (1.6%)	163 (15.8%)	<0.0001
Assisted reproductive technology, *n* (%)	786 (12.7%)	291 (28.2%)	<0.0001
Gestational weight gain, median (IQR)	10.0 (5.0–13.0)	10.0 (6.0–15.0)	0.006
Cervical dilatation at admission (cm), median (IQR)	3.5 (2.5–5.0)	3.5 (3.5–3.5)	<0.0001
PROM, *n* (%)	1081 (17.5%)	80 (7.8%)	<0.0001
Induction of labor, *n* (%)	1823 (29.5%)	284 (27.5%)	0.22
Oxytocin use in labor, *n* (%)	3333 (53.9%)	605 (58.7%)	0.004
Epidural anesthesia, *n* (%)	4709 (76.1%)	452 (43.8%)	<0.0001
Intrapartum fever, *n* (%)	161 (2.6%)	131 (12.7%)	<0.0001
Hemoglobin, median (IQR)	12.2 (11.8–12.6)	12.2 (11.6–12.9)	0.17
WBC, median (IQR)	11.4 (10.2–12.6)	11.4 (9.9–13.9)	0.0003
Platelet count, median (IQR)	209 (188–236)	209 (174–247)	0.36
Neutrophil-to-Lymphocyte Ratio (NLR), median (IQR)	4.1 (3.4–4.8)	4.1 (3.3–5.8)	<0.0001
Birthweight (grams), median (IQR)	3355 (3060–3630)	3150 (2630–3543)	<0.0001
Newborn sex (male), *n* (%)	3125 (50.5%)	578 (56.1%)	0.001

*p*-values are descriptive and were not adjusted for multiple comparisons.

## Data Availability

The data presented in this study are available on request from the corresponding author. Public access is restricted due to ethical considerations and the inclusion of confidential patient data.
